# Prevention of exacerbations in patients with COPD and vitamin D deficiency through vitamin D supplementation (PRECOVID): a study protocol

**DOI:** 10.1186/s12890-015-0101-4

**Published:** 2015-09-23

**Authors:** Rachida Rafiq, Floor E. Aleva, Jasmijn A. Schrumpf, Yvonne F. Heijdra, Christian Taube, Johannes MA Daniels, Paul Lips, Pierre M. Bet, Pieter S. Hiemstra, André JAM van der Ven, Martin den Heijer, Renate T. de Jongh

**Affiliations:** Department of Internal Medicine and Endocrinology, VU University Medical Center, Amsterdam, The Netherlands; Department of Pulmonology, Radboud University Medical Center, Nijmegen, The Netherlands; Department of Pulmonology, Leiden University Medical Center, Leiden, The Netherlands; Department of Pulmonology, VU University Medical Center, Amsterdam, The Netherlands; Department of Clinical Pharmacology and Pharmacy, VU University Medical Center, Amsterdam, The Netherlands; Department of Internal Medicine, Radboud University Medical Center, Nijmegen, The Netherlands

**Keywords:** Chronic obstructive pulmonary disease, Exacerbation, Vitamin D, Immunomodulation, Physical function, Randomised controlled trial

## Abstract

**Background:**

Vitamin D is well known for its function in calcium homeostasis and bone mineralisation, but is increasingly studied for its potential immunomodulatory properties. Vitamin D deficiency is a common problem in patients with COPD. Previous studies have not demonstrated a beneficial effect of vitamin D on exacerbation rate in COPD patients. However, subgroup analyses suggested protective effects in vitamin D deficient patients. Our objective is to assess the effect of vitamin D supplementation on exacerbation rate specifically in vitamin D deficient COPD patients.

**Methods/Design:**

We will perform a randomised, multi-center, double-blind, placebo-controlled intervention study. The study population consists of 240 COPD patients aged 40 years and older with vitamin D deficiency (25-hydroxyvitamin D concentration < 50 nmol/L). Participants will be recruited after an exacerbation and will be randomly allocated in a 1:1 ratio to receive vitamin D3 16800 IU or placebo orally once a week during 1 year. Participants will receive a diary card to register the incidence of exacerbations and changes in medication during the study period. Visits will be performed at baseline, at 6 months and at 12 months after randomisation. Participants will undergo spirometry, measurement of total lung capacity and assessment of maximal respiratory mouth pressure. Several physical performance and hand grip strength tests will be performed, questionnaires on quality of life and physical activity will be filled in, a nasal secretion sample and swab will be obtained and blood samples will be taken. The primary outcome will be exacerbation rate.

**Discussion:**

This study will be the first RCT aimed at the effects of vitamin D supplementation on exacerbation rate in vitamin D deficient COPD patients. Also, in contrast to earlier studies that used infrequent dosing regimens, our trial will study effects of a weekly dose of vitamin D supplementation. Secondly, the immunomodulatory effects of vitamin D on host immune response of COPD patients and underlying mechanisms will be studied. Finally, the effects on physical functioning will be examined.

**Trial registration:**

This trial is registered in ClinicalTrials.gov, ID number NCT02122627. Date of Registration April 2014.

## Introduction

Chronic obstructive pulmonary disease (COPD) is characterized by a persistent airflow limitation and an abnormal inflammatory response of the airways. COPD is predicted to be the third worldwide cause of mortality by 2020 [1]. Exacerbations in COPD determine disease-associated morbidity and mortality [2]. Patients with frequent exacerbations have a more rapid decline in lung function, worse quality of life and decreased exercise performance. Yet, effective treatment alternatives to prevent exacerbations are still lacking.

Vitamin D deficiency is highly prevalent in patients with COPD [3, 4]. Traditionally, vitamin D is associated with bone health. The discovery of the presence of vitamin D receptors (VDR) in many other cells, such as monocytes, macrophages, muscle cells and endothelial cells, has elicited hypotheses of direct vitamin D effects on these cells. These hypotheses are further strengthened by local 25-hydroxyvitamin D-1-alpha-hydroxylase activity, which converts the inactive 25-hydroxyvitamin D (25(OH)D) to the active 1,25-dihydroxyvitamin D (1,25(OH)_2_D), in many of these cells. The presence of vitamin D receptors on immune cells [5] and the high prevalence of vitamin D deficiency among COPD patients has given rise to the hypothesis that vitamin D might have a potential effect in preventing exacerbations in patients with COPD [6].

### Vitamin D and the immune system

There is a large body of evidence being generated *in vitro* and *in vivo* to demonstrate that vitamin D influences the innate and adaptive immune system. 1,25(OH)_2_D is the active form of vitamin D that binds to the VDR, thereby influencing the expression of more than 200 genes. VDR is expressed on a range of immune cells such as macrophages, dendritic cells, and CD4-positive T lymphocytes [5]. In the innate immune system vitamin D modulates Toll-like receptor (TLR)-induced immune responses through inhibition of the NF-κB-pathway and appears to improve antimicrobial defences in general [7, 8]. Vitamin D is capable of inducing endogenous expression of the antimicrobial peptides (AMP) such as cathelicidin. This has been reported in monocytes, macrophages, keratinocytes and in lung epithelial cells [9, 10]. Because AMPs have been found in multiple experimental systems to be essential for defence against a variety of microbial infections, it has been hypothesised that vitamin D can enhance resistance to infections [11]. In addition, vitamin D seems capable of modifying the function of cells classically associated with adaptive immunity whereby activation of VDR downregulates autoimmunity by promoting the differentiation of T-cells into regulatory T-cells [12].

### Vitamin D and pulmonary infections

Vitamin D might influence the development and course of tuberculosis. Patients with low 25(OH)D concentrations have a higher risk of active tuberculosis and vitamin D supplementation may shorten the duration of disease [13]. It is also known that patients with rickets more frequently suffer from airway infections and pneumonia [14]. Several prospective cohort studies in the general population show that lower levels of 25(OH)D are related to increased risk of respiratory infections [15, 16]. A trial with Japanese schoolchildren during the influenza season demonstrated that, compared to placebo, vitamin D supplementation lowered the incidence of influenza A infections [17]. Trials assessing effects of vitamin D supplementation on prevention of respiratory infections in the general adult population have shown conflicting results, which may partly be attributed to differences in prevalence of vitamin D deficiency at baseline and rise of serum 25(OH)D levels during treatments [18, 19].

### Vitamin D and COPD

Patients with COPD are characterised by an abnormal inflammatory response of the airways. Viral and bacterial infections are important triggers of exacerbations and contribute to its progression. Thus, potential effects of vitamin D on the immune system pose an attractive mechanism for the treatment of COPD. Also, in some [20, 21], but not all [22] studies in the general population serum 25(OH)D is positively associated with lung function. Vitamin D deficiency is present in 40–80 % of patients with COPD and is related to disease severity [3, 4]. Recent cohort studies, however, did not show a relationship between 25(OH)D levels and exacerbation rate [23, 24], although these studies had limited statistical power to rule out effects of vitamin D deficiency.

In addition to exacerbations and lung function, skeletal muscle dysfunction in COPD patients contributes to poor exercise capacity, decreased quality of life and increased mortality [25, 26]. In COPD patients, vitamin D deficiency is related to impaired physical performance [27]. In healthy adults, positive effects of vitamin D supplementation have been demonstrated on muscle function and physical performance in particular in older and frail individuals [28, 29].

### RCTs vitamin D supplementation in COPD

Few studies have been performed on the effects of vitamin D supplementation in patients with COPD. In the trial performed by Lehouck et al. [30] vitamin D supplementation did not reduce the incidence of exacerbations. However, in a post-hoc analysis of a subgroup of severely vitamin D deficient patients (25(OH)D concentration < 25 nmol/L), vitamin D supplementation decreased the exacerbation rate. In a very recent multi-center trial by Martineau et al. [31] vitamin D protected against moderate to severe exacerbations in a pre-specified subgroup of vitamin D deficient (25(OH)D concentrations < 50 nmol/L) participants, but not in the study population as a whole.

Two studies have been performed assessing the effect of vitamin D supplementation on physical performance in patients with COPD. A pilot RCT did not show effects of vitamin D supplementation on physical performance, but was limited by the small number of 36 participants and short follow-up of 6 weeks [32]. Also, the study was not specifically aimed at patients with vitamin D deficiency. In the aforementioned RCT by Lehouck et al. a post-hoc subgroup analysis of 50 participants following a rehabilitation programme during the trial was performed [33]. Participants receiving vitamin D supplementation had significantly larger improvements in inspiratory muscle strength and peak exercise tolerance, but not in quadriceps strength and 6-min walking distance. However, this study had limited statistical power and is only applicable for patients following a rehabilitation programme. These findings justify a well-designed RCT to study effects of vitamin D supplementation on muscle strength and physical performance.

Little is known about the total dose and dose interval needed for extra-skeletal effects of vitamin D. In the study of Lehouck et al. [30] participants received a monthly dose of 100.000 IU. In the study of Martineau et al. [31] participants received 120.000 IU every two months. A large dose interval improves compliance but might also cause fluctuating levels of vitamin D metabolites [34]. In two RCTs assessing the effect of vitamin D supplementation on falls and fractures, an increase of fall and/or fracture incidence were shown using annual high dose supplementation [35, 36]. While the mechanism by which vitamin D might cause an increase in falls remains uncertain, several authors suggest it is the dosing interval rather than the total dose that determined these outcomes [37]. These results emphasize the need for an RCT studying a more frequent dosing regimen of vitamin D.

In the present study we aim to study the effects of vitamin D supplementation on exacerbation rate in COPD patients with vitamin D deficiency. In addition, we will also assess the effects of vitamin D on several measures of physical performance. In our trial, we will administer a weekly dosing regimen in contrast to earlier studies, which used larger dosing intervals.

## Methods

### Study design and participants

**Fig. 1:**
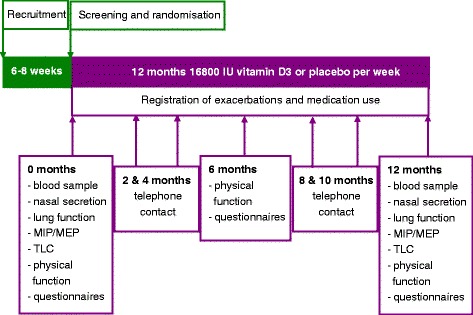
Flowchart of study procedures during the PRECOVID trial. Randomisation will take place within 6-8 weeks after an exacerbation. During the study period of one year three study visits and four telephone contacts will take place. MIP: Maximal inspiratory pressure; MEP: Maximal expiratory pressure; TLC: Total lung capacity

The study is designed as a randomised double-blind, multi-center, placebo-controlled trial, with an intervention (*n* = 120) and a control group (*n* = 120). The study population consists of COPD patients with GOLD stages II-IV. Participants will be included if they have a vitamin D deficiency (25(OH)D concentrations <50 nmol/L) and a recent exacerbation of COPD. The eligibility criteria are described in Table 1. Participants will be recruited if they present with an exacerbation of COPD at the outpatient clinic or emergency ward. Screening serum 25(OH)D concentration will be measured during the exacerbation period. Inclusion and randomisation will take place 6–8 weeks after recruitment or at a later time point as soon as the participant has recovered. An exacerbation is defined as sustained worsening of respiratory symptoms during 48 h requiring oral corticosteroid, antibiotic or combination treatment that was initiated by a physician [38]. Convalescence is defined as recovery to the stable state. The recruitment will start in three academic hospitals and five peripheral hospitals in the surroundings of the academic centers. Study visits will be performed at baseline (t = 0), 6 months (t = 6) and 12 months (t = 12) after randomisation as depicted in Fig. 1. Participants will be contacted by telephone at 2, 4, 8 and 10 months The study is approved by the Medical Ethics Committee of the VU University Medical Center, Radboud University Medical Center and Leiden University Medical Center. Written informed consent will be obtained from all participants.

**Table 1:** Eligibility criteria for the PRECOVID trial

Inclusion criteria	Exclusion criteria
Vitamin D deficiency (serum 25(OH)D < 50 nmol/l)	Severe vitamin D deficiency (serum 25(OH)D <15 nmol/l),
Postbronchodilator FEV_1_/FVC < 0.70, FEV_1_ < 80 % and diagnosis COPD confirmed by a physician	Use of a supplement containing more than 400 IU vitamin D per day
Recent COPD exacerbation	Use of maintenance dose oral corticosteroids
≥10 packyears of smoking	Diagnosed asthma
Age ≥ 40 years	Diagnosed osteoporosis
Written informed consent	Self-reported history of hypercalcaemia or nephrolithiasis
Ability to comply with all study requirements.	Self-reported presence of sarcoidosis
	Diagnosed chronic kidney disease stage 4 or higher (eGFR ≤ 29 ml/min/1.73 m2)
	Interfering malignant diseases
	Life expectation of less than 1 year on the basis of concurrent disease
	Current participation in a clinical rehabilitation program
	Pregnant or lactating women, or subjects who intend to become pregnant within the study period
	Potentially unreliable patients and those judged by the investigator to be unsuitable for the study
	Serious mental impairment i.e. preventing to understand the study protocol or comply with the study aim

*25(OH)D* 25-hydroxyvitamin D, *FEV*_*1*_ Forced Expiratory Volume in one second, *FVC* Forced Vital Capacity, *eGFR* estimated Glomerular Filtration Rate with the MDRD formula

### Intervention

The participants will receive either vitamin D 16800 IU (3 tablets of 5600 IU) or a matching placebo (3 placebo tablets) orally once a week during 12 months in accordance with the randomisation. The study medication will be prepared under supervision of the clinical pharmacy of the VU University Medical Center. Participants will be asked to return the study medication that is left over and these tablets will be counted as a measure for compliance. Participants are allowed to use a maximum of 400 IU vitamin D a day during the study, as this is the recommended daily intake by the Dutch Health Council. Participants will be asked about their usual intake of dairy products. Participants are advised (according to the advice of the Dutch Health Council) to increase their intake to a level corresponding with 1000 mg calcium per day. If this is not feasible, it will be advised to use calcium supplements to a total calcium intake of 1000 mg/day during the study.

### Randomisation and masking

For treatment allocation in this trial, we will apply the sequential balancing method with study center as the first step in the balancing algorithm, followed by gender, age and current smoking. These variables are (potential) prognostic factors and thus should be balanced over the treatment arms. Pharmacists of the VU University Medical Center, who are independent from the clinical research team, will randomly assign participants by using a computer-generated randomisation list and will prepare the study medication. After the last participant finishes the trial masking will continue until after the data analyses.

## Outcomes

### Primary outcome

#### Exacerbation rate

Primary outcome is exacerbation rate. An exacerbation is defined as sustained worsening of respiratory symptoms during 48 h requiring oral corticosteroid, antibiotic or combination treatment that was initiated by a physician. Respiratory symptoms include at least one of the Anthonisen criteria, which are increased dyspnoea, sputum volume or sputum purulence, with or without minor symptoms such as cough, fever, common cold, wheezing or sore throat [38]. Participants will register symptoms and changes in medication on a diary card during the whole study period. During each telephone contact and study visit diary cards will be discussed and reviewed. Also questions will be asked regarding hospitalization during the last months. If necessary, information on changes in medication and COPD exacerbations during the study period will be confirmed through contact with the providing pharmacist, the general practitioner and hospital.

### Secondary outcomes

#### Time to first and second exacerbation and time to fist hospitalisation

Time to first and second exacerbation and time to first hospitalisation will be registered on the basis of the diary card and interviews.

#### Use of antibiotic and corticosteroids

With the use of earlier mentioned diary card and interviews the use of antibiotics and corticosteroids will be registered.

#### Spirometry measures

At t = 0 and t = 12 participants will undergo spirometry. The spirometry will be performed according to American Thoracic Society/European Respiratory Society (ATS/ERS) guidelines [39]. Only post-bronchodilator forced expiratory volume in one second (FEV_1_) and forced vital capacity (FVC) will be determined. Measurements will take place after inhalation of the sympathomimetic salbutamol and/or the anticholinergic ipratropium bromide. Inspiratory capacity (IC) and expiratory reserve volume (ERV) will also be determined according to ATS/ERS guidelines [39].

#### Lung volumes

Measurements of absolute lung volume, residual volume (RV), functional residual capacity (FRC) and total lung capacity (TLC) will be assessed by body plethysmography according to guidelines of the ATS/ERS task force at t = 0 and t = 12 [40, 41].

#### Maximal respiratory mouth pressures

Respiratory muscle strength will be tested by measurement of the maximal inspiratory and expiratory mouth pressure (MIP and MEP, respectively). Measurement will be made with a mechanical pressure gauge that is connected to a mouthpiece according to the revised ATS guideline [42].

#### Physical performance

During every visit (t = 0, t = 6 and t = 12), participants will perform physical performance tests.

In the 6-min walking test the participant is asked to walk as far as possible during a period of 6 min. The participant will walk up and down a hallway. The 6-min walking test is performed according to the ATS guidelines [43].

In the chair-stands-test the participant will be asked to fold his arms across his chest and to stand up from a sitting position and sit down five times as quickly as possible [44, 45].

In the 3-meter walking test the participant is asked to walk three meters, turn around 180° and walk back [44, 46].

In the tandem test the participant is asked to stand with the heel of one foot directly in front of, and touching the toes of, the other foot for at least 20 s with his eyes open and closed [44, 47].

#### Muscle strength

Muscle strength will be assessed with measurement of hand grip strength. The participant is seated in a chair and holds the dynamometer in the hand, with the arm at right angles and the elbow by the side of the body. The handle of the dynamometer is adjusted if required. When ready, he squeezes the dynamometer with maximum isometric effort, which is maintained for about 5 s. No other body movement is allowed. This measurement will be performed three times with both hands [48].

#### Quality of life

Quality of life will be assessed by three questionnaires These will be completed at every study visit.

The St. George’s Respiratory Questionnaire (SGRQ) is designed to measure health impairment in patients with asthma and COPD [49, 50] and produces a symptoms score and an activity and impacts score.

The Short Form 12 health Survey (SF-12) is a multipurpose short-form generic measure of health status [51]. It is a validated subset of the larger SF-36 and monitors health in general and in specific populations. These scores correlate highly with the SF-36 versions [52]. The SF-12 has been previously used in patients with COPD [50, 53].

The Clinical COPD questionnaire (CCQ) is a simple 10-item, health-related quality of life questionnaire with good psychometric properties [54]. The questionnaire is responsive to pulmonary rehabilitation and a minimal clinically important difference has recently been defined [55].

#### Anxiety and depression

Anxiety and depression will be assessed by means of two questionnaires and will be completed at every study visit. The Hospital Anxiety Depression Scale (HADS) is a questionnaire measuring feelings of depression and anxiety [56]. Reliability and validation of the Dutch translation of the HADS has been reported [57]. The HADS is frequently applied in research in patients with COPD [58, 59].

The Center for Epidemiologic Studies Depression Scale (CES-D) is a self-report scale designed to measure depressive symptoms in the general population. The development of the scale has been described in detail elsewhere [60]. The scale has been used in several COPD populations [61, 62].

#### Physical activity

Physical activity will be assessed by the Short QUestionnaire to ASses Health enhancing physical activity (SQUASH). SQUASH is a self-report instrument to measure habitual physical activity [63]. The questionnaire has been used in COPD populations [64].

#### Antimicrobial peptides and pro-inflammatory mediators in nasal secretions

Nasal secretions will be collected by vacuum-aided suction to prevent dilution that occurs when using lavage-based methods [65]. The nasal secretions will be analysed for the presence of antimicrobial peptides and pro-inflammatory cytokines (hCAP18/LL-37, HNP1-3, NGAL, IL-1ß, TNF-α, IL-6) using enzyme-linked immunosorbent assays (ELISA).

#### Typing of bacteria and viruses in nasal secretions

A nasal swab will be obtained using standard procedures. PCR on presence of bacteria and respiratory viruses (non-typeable *Haemophilus influenzae*, *Streptococcus pneumoniae*, *Moraxella catarrhalis* and a panel of respiratory viruses including rhinovirus) will be performed.

#### Inflammation and host response against infectious agents

In one of the academic centers peripheral blood mononuclear cells (PBMC) and platelets will be derived from 50 participants before and after the intervention. Venous blood will be drawn in EDTA and 3.2 % sodium citrate tubes after which PBMC and platelet-rich plasma (PRP) will be isolated, washed and suspended. PBMC will be stimulated with various agonists to TLR (bacterial, fungal and viral) and with microbial stimuli. The following stimuli will be used: RPMI (control), LPS (TLR4), Pam3Cys (TLR2), Poly (I:C) (TLR 3), *Streptococcus pneumoniae, Non-typeable Haemophilus influenza, Pseudomonas aeroginosa, Aspergillus fumigatus*, sonicated *Mycobacterium avium* and *Candida albicans*. Pro- and anti-inflammatory cytokines will be measured before and after stimulation: TNF-α, Il-1ß, IL-6, IFN-γ (24 h incubation) and IL-10, IL-17 and IL-22 (7 days incubation). All cytokines will be measured in cell culture supernatants using ELISA. PRP will be used for analyses of platelet function in terms of activation and aggregation. Second, phenotyping of circulating immune cells will be done using advanced multiparameter flowcytometry and functional readouts. A whole blood staining will be used to be able to calculate absolute numbers of CD4 (including Treg), CD8 T cells, NK cells, B cells and monocytes. Next, a detailed phenotypic analysis of the T cell compartment will be performed by additional staining of whole blood or isolated PBMC using antibodies. Functional characteristics of the T cell compartment (CD4+, CD8+, CD4+/Foxp3+ (Treg)) with respect to cytokine production (IFN-γ, IL-2, IL-4, IL-5, IL-10 and IL-17A) and their associated transcription factors (Tbet, Gata-3, RORyt) will be analysed.

### Sample size calculation

The study is designed to demonstrate a minimum difference of one exacerbation per patient-year between the vitamin D and the placebo group. We based our assumptions on post-hoc analyses of the RCT by Lehouck et al.[30], which had a similar patient sample, although without selection for vitamin D deficiency. Based on differences in Poisson means, a sample size of 94 participants per group is needed to demonstrate a difference of 1 exacerbation per patient-year with 80 % power at 5 % significance. This number was based on the assumption of 2.45 exacerbations per patient-year in the intervention group and 3.45 in the placebo group. To account for a dropout rate of 20 % 120 participants will be recruited per study group.

### Statistical analysis

#### Primary outcome

The primary outcome of the trial, exacerbation rate, will be analysed with generalized linear models for a Poisson distribution. Analyses will be adjusted for the variables included in the balancing algorithm for randomisation, i.e. study center, gender, age and current smoking. The outcomes will be presented as rate ratios with their 95 % confidence interval.

#### Secondary outcomes

Time to first exacerbation and hospitalisation will be assessed with Kaplan–Meier curves and Cox proportional hazard models. Dichotomous secondary outcomes will be analysed with logistic regression models and continuous secondary outcomes with analysis of covariance. All analyses will be adjusted for the variables included in the balancing algorithm for randomisation, i.e. study center, gender, age and current smoking. Because of the exploratory nature of the analyses, no correction will be made for the multiple analyses. All analyses of the trial will use the intention-to-treat population, defined as all randomly assigned participants who received at least 1 dose of study medication. A per-protocol analysis will be performed on those participants with a good compliance i.e. obtaining a serum 25(OH)D concentration above the 20^th^ percentile after 12 months. Also, a subgroup analysis will be performed in participants with a baseline serum 25(OH)D concentration of 25 nmol/L or less.

## Discussion

This RCT is the first intervention study examining effects of vitamin D supplementation in a cohort of vitamin D deficient COPD patients. With this study we expect to gain more insight in the effects of vitamin D supplementation on exacerbation rate and both pulmonary and physical function. The two previous RCTs [30, 31] did not show an effect of vitamin D supplementation in the total study population of COPD patients. They did, however, show a protective role of vitamin D against exacerbations in deficient patients. These are promising results and emphasise the need for an RCT specifically in vitamin D deficient patients considering the high prevalence of vitamin D deficiency in patients with COPD. In our study participants with a severe vitamin D deficiency (25(OH)D <15 nmol/L) will be excluded because of ethical considerations.

Our study differs in several other aspect from previous studies. In addition to the effects of vitamin D on exacerbation rate, present study will focus on potential underlying mechanisms. In nasal secretion the presence of antimicrobial peptides and pro-inflammatory mediators will be measured and the microbial composition of the nasal mucosa will be analysed. Furthermore, PBMCs from a subgroup of participants will be challenged with TLR-ligands and several pathogens in order to measure cytokine responses. These tests will be performed before and after the intervention to measure effects ofvitamin D supplementation on cytokine responses and therefore inflammation and host respone. Finally we will assess physical performance in several different manners.

In contrast to earlier studies, we will use a weekly dosing regimen, which leads to more stable 25(OH)D levels [34], but may influence compliance. This is important since little is known about the total dose and dose interval needed for extra-skeletal effects of vitamin D.

As stated earlier exacerbations have a major impact on somatic and mental health status of COPD patients [2]. With the increasing prevalence of COPD, the economic burden will expand significantly [1]. Exacerbations constitute the most important direct costs associated with COPD. Treatments directed to reduce incidence and severity of exacerbations improve long-term health status and reduce health care costs [66]. Vitamin D might therefore be an attractive treatment alternative, as it is easily applicable, cheap and safe.
